# Co-creating learning: A qualitative exploration of physiotherapy clinical education

**DOI:** 10.4102/sajp.v82i2.2299

**Published:** 2026-04-30

**Authors:** Alison Lupton-Smith, Anna M. Schmutz

**Affiliations:** 1 Department of Health and Rehabilitation Sciences, Faculty of Medicine and Health Sciences, Stellenbosch University, Cape Town, South Africa; 2 Department of Health Professions Education, Faculty of Medicine and Health Sciences, Stellenbosch University, Cape Town, South Africa

**Keywords:** clinical education, workplace-based learning, physiotherapy education, agency, learning co-creation

## Abstract

**Background:**

A large part of physiotherapy clinical training is situated in the workplace. Learning in an authentic environment is supported by facilitators.

**Objectives:**

This study focussed on the perceptions and understanding of physiotherapy students and facilitators while learning on the clinical platform.

**Method:**

A qualitative methodology within an interpretivist paradigm was followed using convenience sampling.

**Results:**

Physiotherapy students (*n* = 45) in their fourth and final year of study and clinical facilitators of this student group (*n* = 13) were invited to participate in a focus group discussion (FGD) for the students (*n* = 7) and semi-structured interviews for the facilitators (*n* = 7). An inductive data analysis approach was followed. Three themes were generated: *Learning as a Shared, Active Process*; *Relationships, Role Modelling and Power Dynamics in Clinical Learning*; and *the Clinical Environment as Both Constraint and Catalyst*.

**Conclusion:**

Across themes, learning was shaped by how students and facilitators understood and enacted their roles, the degree to which student agency was recognised and supported, and how relational safety and environmental constraints mediated engagement. Implicit and sometimes misaligned expectations between students and facilitators influenced whether learning opportunities were taken up or constrained. Clinical learning emerged as a co-constructed, relational process rather than a unidirectional transfer of knowledge.

**Clinical Implications:**

Supporting meaningful learning in the clinical environment requires greater attention to implicit expectations, relational dynamics and the conditions under which student agency can be enacted.

## Background

The clinical environment provides an authentic learning context where physiotherapy students transition from theory to practice, developing skills necessary to function as healthcare professionals. Unlike classroom or simulated settings, workplace-based education is dynamic, complex and unpredictable, requiring students to apply prior learning while adapting to real-world demands (Dornan, Scherpbier & Boshuizen 2009; Embo et al. 2014).

Multiple studies have explored factors influencing student learning across educational contexts, including physical, social, organisational, cultural and educational dimensions (Bordage & Harris 2011; Genn 2001; Gruppen et al. 2018; Karani 2015). Recommendations to enhance learning experiences often emphasise structured programmes, strong relationships with educators and accessible support (Henning et al. 2011; Karani 2015). Central to these approaches is active engagement, in which students take ownership of their learning (Billett 2004). Dornan et al. (2009) argue that active participation enables medical students to ‘learn how to learn’, a vital competency for both academic and clinical environments.

Health professions education (HPE) increasingly promotes active learning strategies, positioning students as co-creators of their education (Ramnanan & Pound 2017). However, research highlights ongoing challenges: students frequently rely heavily on facilitators for direction, and often feel underprepared to apply theoretical knowledge in clinical practice (Berkhout et al. 2018; Dornan et al. 2005; Prince et al. 2005). While prior studies have examined barriers and facilitators of clinical education, less attention has been paid to how students and educators themselves conceptualise learning, their roles within this process and what this means for engagement in workplace-based settings.

Understanding these perceptions is critical for informing programme design and supporting more effective learning in clinical education. This study therefore investigates how physiotherapy students and clinical facilitators (CF) experience and perceive learning in the clinical context and their respective roles.

## Research methods and design

A qualitative methodology within an interpretivist paradigm was used to gain an understanding of physiotherapy students’ and facilitators’ perceptions and understanding of learning (De Vos et al. 2011).

### Population and sampling

Convenience sampling was used, whereby physiotherapy students (*n* = 45) in their fourth and final year of study and selected CF of this student group (*n* = 13) at Stellenbosch University (SU), Cape Town, South Africa, were invited to participate in the study. Fourth-year students were chosen as they had the most exposure to clinical learning at the time of the study, which took place in the first semester of their final year. The CF represent qualified physiotherapists who are appointed to facilitate students’ learning in the workplace. Clinical facilitators have varying levels of clinical and supervision experience and areas of expertise. Clinical facilitators supervise students weekly for 1.5 h per week. Clinical learning takes place in the Cape Metropole and surrounding areas across levels of healthcare service.

Potential participants were invited to the study through an email detailing the study was distributed by the research assistant, and those who volunteered and provided informed consent were included in the study.

### Data generation

Data were generated by means of a focus group discussion (FGD) for the students (*n* = 7) with the purpose of getting a collective sense of their understanding of learning. Semi-structured interviews were employed for the facilitators (*n* = 7), to provide for possible different understanding based on the varied years of experience in supervising (Du Plooy-Cilliers, Davis & Bezuidenhout 2014). A discussion guide with open-ended questions was used in the FGD and interviews, respectively (Appendix 1). The FGDs and semi-structured interviews were conducted by a research assistant familiar with the study, audio-recorded and then transcribed by the research assistant in preparation for analysis.

### Data analysis

An inductive data analysis approach was followed using the six-phase thematic analysis framework as introduced by Braun and Clarke (2021), for both the FGDs and individual interviews (Braun & Clarke 2021). ATLAS.ti software version 24 (ATLAS.ti, Scientific Software Development GmbH, Germany) was used to assist with the data analysis process. Verbatim transcripts were studied to familiarise the researchers with the data. The data were coded independently by two researchers, who then met to discuss and refine the codes to enrich the interpretation. Patterns within the codes were used to develop themes.

To ensure credibility and confirmability, peer examination was implemented by discussing the research process and findings with experts not involved in this study. To facilitate transferability and dependability, the sampling and findings from this study were discussed in detail with all researchers involved in the study, while data analysis was conducted as an iterative process (Frambach, Van der Vleuten & Durning 2013).

It is acknowledged that the researchers’ roles as educators and, in one case, clinical co-ordinator (Anna M. Schmutz) may have introduced power relationships that could have influenced both CF’s and students’ participation and responses. To mitigate this, the researchers were not involved in any data generation and engaged only in the analysis of de-identified data. The researchers are both white female academics who work as lecturers (Anna M. Schmutz, Alison Lupton-Smith) and clinical supervisors (Alison Lupton-Smith) within the physiotherapy programme. While this professional positioning did not influence data collection, it nonetheless shaped the analytic lens through which the data were interpreted. Ongoing reflexivity was therefore required and used to interrogate assumptions about learning, supervision and student agency throughout the analytic process.

### Ethical considerations

Ethical clearance to conduct this study was obtained from the Social, Behavioural and Education Research (SBER) (REC-2021-23954), and institutional approval (IG-3062) was obtained from Stellenbosch University (SU), as well as approval from the Undergraduate Programme Committee of the Division of Physiotherapy for the study. All participants provided informed consent.

## Results

Seven female students took part in the FGD. Seven clinical educators (one male) participated in individual interviews. Two clinical educators were full-time employed lecturing staff. The mean (standard deviation [s.d.]) years of experience in clinical education was 12 (s.d. 7) years, with a range of 4–22 years.

Three themes were developed from the data, which spoke to learning in the clinical environment. The first theme, Learning as a shared, active process, speaks to students’ and facilitators’ understanding and experience of learning. The second theme, relationships, role modelling and power dynamics in clinical learning, speaks to how the positionality and attributes of people contribute to learning in the clinical environment. Lastly, theme three speaks to the clinical environment as both constraint and catalyst, the physical and non-physical spaces in which learning takes place and the contribution of these spaces to learning.

### Theme 1: Learning as a shared, active process

This theme captures how students and facilitators experienced learning as active and shared, while revealing tensions around preparedness, responsibility and competing expectations of who ultimately drives learning in the clinical environment.

Both students and CF saw the clinical environment as a place where students had the opportunity to apply and integrate their theoretical knowledge. Facilitators viewed preparedness as an implicit expectation for learning in the clinical environment:

‘I think clinical learning is taking all this theory and practical that you’ve done on your own and on healthy human beings and then applying it to the real world and people with real diagnoses and trial and error.’ (FGD1)

‘When you when you reach the clinical setting, the understanding is that I’m not going to teach you the basics.’ (CF6)

Students showed a nuanced perspective on preparedness. Some described tension in navigating the implicit expectations of knowledge and competence as clinicians in training and their ongoing identity as learners. Preparedness appeared to enable students to engage with uncertainty and step beyond their comfort zones, recognising what they already know and still can learn:

‘[*T*]his is actually a big problem for us as we think that we should know what’s going on when we go to clinical, but clinical is still a module and it is a place of learning. So, I think we’re often intimidated by the fact that we should know everything. We have real people in front of us but that’s an experience that that you learn from whether it’s good or bad or dangerous it is a learning opportunity well.’ (FGD1)

‘[*A*]ctive process that is actually involved and I think learning is also stepping out of comfort zone because you can’t learn with what you already know.’ (FGD1)

The importance of preparedness, on the part of a student, was amplified by the added responsibility of having a patient or person who is now dependent on them. This added responsibility was also noted by facilitators as influencing learning. Furthermore, the fluid nature of the clinical environment, where no patient is the same, necessitated a heightened preparation, responsibility and activation towards learning the part of the students:

‘[*L*]earning is recognising that it’s almost a responsibility … But when we’re dealing with very sick people or just people with health conditions, if I don’t show up knowing a certain amount, I’m actually putting them at risk. Whether that’s life and death or just a decreased functional level full and decreased quality of life. If I don’t show up as best as I can with what I know and I’m impacting somebody’s life.’ (FGD1)

‘[*L*]earning is a whole process and I think an ongoing process because things change constantly. People are different. So in the clinical platform you’re never going to get the same patient. … So you’re constantly learning because things are constantly changing.’ (FGD1)

Though students frequently and explicitly described themselves as active learners, their accounts revealed divergent expectations of facilitation. For some, active learning depended on facilitators recognising and responding to learning bids; for others, facilitators were expected to actively initiate and drive learning encounters:

‘[*P*]eople of authority who are in the clinical environment who are passive and in the sense that I can come to them with what I know, which I think is our responsibility. And then they still don’t take that or use the opportunity to teach us or show as well. So I think they need to be active in that role.’ (FGD1)

‘I think what is the assumed or ideal is that our supervisors would facilitate [*learning*]. So a person from the university that we know, a lecturer or an outsider, I don’t actually know why they get employed. But, hey would supervise the session that either you lead, or, which is ideal, or they lead.’ (FGD1)

Most facilitators also alluded to the active role of students in that they viewed their role as being to guide students; however, this assumed student agency was seldom made explicit. Some facilitators recognised the importance of facilitating thinking in students rather than imparting knowledge, while others saw their role as primarily imparting knowledge:

‘My purpose was more to facilitate the student to reason this out.’ (CF7)

‘So I’m very strong in encouraging them to have a specific objective and not just you know, looking at me to take the lead.’ (CF8)

‘I can go on and on and on with the students, but they became sort of tired of the same, you know, the same area and things like that.’ (CF9)

While other facilitators showed restraint or awareness on their part, such as when to hold back on giving input to allow the students the opportunity to discover the learning for themselves or to include the patient in the learning process:

‘I try and keep myself quiet, but normally it’s also kind of like if there is something and you’ll, discuss it in in front of the patient as well where they are also part of learning.’ (CF10)

‘The patient, I think, is this the biggest learning opportunity is the patient because no two patients are the same.’ (CF8)

Entrenched practices within clinical environments limited opportunities for adaptation to learning:

‘It’s the well … we’ve done it just there for millions of years.’ (CF10)

Discomfort functioned as a productive site of learning; however, whether discomfort translated into learning depended on how the individuals engaged with uncertainty, mistakes and co-learning:

‘Like no one likes the feeling of not knowing but if you put yourself in that vulnerable position, that is what learning is.’ (FGD1)

‘I didn’t realise that learning would come so much from making mistakes and I think mistakes are hectic, but it could be small things in terms of your reasoning like, I didn’t think about that, that’s a mistake. I should actually think like this.’ (FGD1)

‘[*W*]hen they come up with stuff that they’ve read or engaged with, and then I’m able to say, OK, well, that’s something new. I didn’t know about. Thank you for that.’ (CF8)

This suggests that discomfort alone did not generate learning; rather, learning on the part of both student and facilitators was manifested through relational engagement with the uncertainty.

### Theme 2: Relationships, role modelling and power dynamics in clinical learning

This theme examines how relationships, role modelling and power relations shaped students’ access to learning opportunities and willingness to engage in learning in the clinical environment.

Relational safety enabled students to take learning risks, including asking questions and making mistakes; in its absence, students found relational safety in peer learning:

‘So I think the way you approach someone will also depend on you know understanding where they’re at. So a little bit of like compassion.’ (FGD1)

‘other people come to us like the clinicians or people in authority ask us we feel so belittled … you know we shouldn’t take it that way, but you, you, you know you do. So that’s why peer learning just works well.’ (FGD1)

While students emphasised interpersonal relationships, a broader, system perspective of the importance of relationships was highlighted by facilitators:

‘I think if you acknowledge them and you greet them [*clinicians*], you start forming a relationship. So, for me it’s easier to do that, but for the students it’s really challenging.’ (CF7)

Through role modelling, students learned not only clinical practices, but also norms about communication, authority and professional behaviour, which shaped how they engaged in clinical spaces:

‘I’d appreciate if people would just set the example because I think you learn a lot more from seeing what people do versus what they say. You know the whole practice vs preach kind of thing. So I think their role is to set an example and that’s not necessarily when I’m in a session with them. That’s with every single patient.’ (FGD1)

How individuals positioned themselves within the learning engagement impacted the learning. Confidence, even in the presence of doubt, enabled students to engage with learning opportunities and navigate hierarchical relationships:

‘So, like you go in a bit more confident but you’re still very unsure, but the fact that you just went says the lot like that’s how you actually learn, because like you said the way they approach you, so you need to learn that not everyone can always be nice.’ (FGD1)

Facilitators alluded to the students’ confidence and self-efficacy as an important contributing factor to their learning in the clinical environment. In some cases, facilitators described how their confidence and perceived self-efficacy in the facilitation role influenced how they engaged with students and learning opportunities:

‘I’m also always worried that. I’m not. I’m not giving them enough info information is a missing out on something in that hour.’ (CF9)

### Theme 3: The clinical environment as both constraint and catalyst

This theme examines how characteristics of the clinical environment both constrained and enabled learning, influencing how students and facilitators engaged with learning opportunities.

The affordances for learning offered by the clinical environment were shaped by participants’ perspectives and their perceived agency within the learning context:

‘I think about using your environment and the resources that you have, because I think that we have so many resources.’ (FGD1)

‘… in at the deep end of it because it’s foreign environment, it’s new patients, it is different superiors, different clinicians that are expecting things of them from where they are coming in terms of being undergraduate students.’ (CF7)

Limited resources necessitated creativity and innovation, allowing students to enact their agency in their learning. While physical resources were often limited, the availability of human resources – including medical professionals, peers and patients – proved valuable to students:

‘[*A*] lack of resources that sparks learning to whether asking how would you actually get someone upright if not with the Tilt Table … So, learning in yourself, thinking creatively.’ (FGD1)

‘Learning is very patient facilitated.’ (FGD1)

Inherent context and system norms, such as differences between university expectations and clinical practices, were experienced as barriers to learning:

‘I do sometimes feel the clinicians are a barrier to learning where they do your things a certain way where we as a university do something in a different way.’ (CF10)

‘… but I think the power dynamics stay in place.’ (CF6)

Competing service delivery demands at clinical sites posed challenges to learning, where students needed to navigate a dual role of service provider and learner. These demands fostered adaptability in students:

‘Patients are sometimes not long in hospitals, so students don’t have a lot of time to reason things out, they have to get the patient up and going and get home. So that places them under quite a bit of pressure and it might be a very new experience for them, and they might be feeling quite uncertain and sometimes threatened in that type of a situation because of the pressure patient must be outpatient must be discharged.’ (CF7)

Together, these findings suggest that the clinical environment shaped learning opportunities in ways that depended on how participants perceived and enacted their agency within environmental constraints.

## Discussion

Learning in the clinical environment occurs through a complex interplay of factors mediated by agency. Learning was seen as an active process; however, the degree to which this process was enacted was dependent on the stance each individual took and how individuals positioned themselves within the learning engagement. Underpinning the learning engagement and positionality of individuals was the complexity of the environment in which the learning took place.

While the clinical environment consistently provides an authentic learning space, the way that students and facilitators enter and engage with it shapes the environment’s potential for learning (Berkhout et al. 2018; Billett 2001). Tensions existed within and between the individuals as they navigated the complexity of the environment and their place in it. The findings suggest that these tensions could be perceived as either barriers or facilitators to learning in the clinical environment, depending on the stance that is taken by the individual (Berkhout et al. 2018). A more negative stance – characterised by defensiveness, rigid adherence to established practices or a focus on constraint – may narrow engagement and limit learning opportunities. In contrast, a more positive stance – marked by curiosity, openness to uncertainty and willingness to engage relationally – appeared to enable individuals to reframe challenges as opportunities for growth. Despite the challenge of navigating an environment with competing demands, and implicit and inconsistent expectations, students often saw learning opportunities in what may have otherwise been perceived as barriers. This suggests that students approached the clinical environment through a wider lens, attending not only to formal teaching moments, but also to relational interactions, contextual constraints and unplanned experiences as potential sites of learning (Sheehan et al. 2017). Students seemed to have entered learning engagements with a stance of curiosity, allowing themselves the opportunity to uncover learning opportunities in the clinical environment and enact their agency.

Students’ accounts in this study demonstrated a clear sense of active participation in learning, aligning with previous contextual studies that position learners as agents within the clinical environment (Ernstzen, Bitzer & Grimmer-Somers 2010). This finding contrasts with earlier literature emphasising students’ dependence on facilitators for learning direction and knowledge acquisition (Dornan et al. 2005). Our findings suggest a shift towards greater student agency within clinical learning contexts, potentially reflecting evolving learning approaches that emphasise facilitation rather than instruction (Ernstzen et al. 2010). Furthermore, students in this study tended to recognise learning as extending beyond the acquisition of specific hands-on skills, encompassing relational engagement, professional behaviours and learning from a range of experiences (Burgess et al. 2020).

Facilitators, in contrast, more often focussed on discrete learning tasks and skill development, with some positioning themselves as central to the learning process. Within this framing, student agency was not always explicitly recognised. It is important to note that facilitators in this study were employed primarily for student education rather than service delivery. This may partially explain differences in how roles were assumed and how learning was prioritised within clinical encounters.

The positioning of facilitators as central to the learning process appears to sit in tension with the value both students and facilitators placed on adaptability and flexibility in learning. When facilitators are positioned as central to the learning process, opportunities for students to independently recognise and act on emergent learning moments may be constrained. Self-regulated learning involves not only engagement with planned learning activities, but also the capacity to recognise and act on emergent learning opportunities within the clinical environment, which requires flexibility and adaptability (Berkhout et al. 2018). Students in this study demonstrated an appreciation of this aspect of learning, recognising the importance of adapting to context and taking up learning opportunities as they arose.

While students demonstrated an emerging capacity for self-regulated learning (SRL), our findings suggest that the enactment of SRL in the workplace was not solely an individual endeavour but was mediated through relationships and contextual dynamics (Berkhout et al. 2018; Dornan et al. 2009). Participation in communities of practice required relational access, particularly relationships with facilitators and clinicians who could legitimise students’ participation. Students emphasised the importance of being recognised as whole persons rather than merely as ‘students’, which appeared to shape their willingness to engage, ask questions and take learning risks (Telio, Regehr & Ajjawi 2016). However, implicit and sometimes misaligned expectations regarding roles and responsibilities risked constraining these learning opportunities (Telio, Ajjawi & Regehr 2015), highlighting the need for more explicit negotiation of expectations to support relationally mediated SRL.

The concept of the educational alliance provides a useful lens through which to interpret these findings. Similar to reports in medical and postgraduate education (Bowen, Marshall & Murdoch-Eaton 2017; Telio et al. 2015, 2016), learning in this study appeared to be co-created through interactions between students and facilitators, rather than delivered unidirectionally (Boud & Molloy 2013). This alliance is underpinned by trust and relational engagement (Telio et al. 2016), both of which were reflected in students’ descriptions of the need for a ‘safe space’ for learning. While the notion of a safe space remains contested, in this context it appears to reflect a constructive learning environment in which students felt able to express uncertainty, make mistakes and engage with discomfort (Dornan et al. 2009; Ernstzen et al. 2010; Hardie et al. 2022; Molloy et al. 2025). Importantly, our findings suggest that such environments support a relational form of SRL, in which students’ capacity to regulate their learning is shaped by trust, power relations and contextual affordances rather than individual effort alone (Berkhout et al. 2018; Dornan et al. 2009).

### Limitations and future directions

This study has several limitations related to sampling and participant representation. A convenience sampling approach was used, and data saturation may therefore not have been achieved, which may have limited the breadth of perspectives captured. However, the study prioritised analytic depth and contextual understanding over exhaustive representation, and the findings should be understood as theoretically informative rather than statistically generalisable, with transferability dependent on reader judgement. In addition, all participants were female, limiting exploration of how gender may shape experiences of learning, agency, confidence and power in the clinical environment. Academic performance data were not collected, and it is therefore unclear how variations in academic standing may have influenced participants’ engagement with learning. Given the relational and positional nature of learning highlighted in this study, future research should include more gender- and performance-diverse samples to examine how these dynamics may differ across groups.

A further limitation is that facilitators included in this study were not clinicians involved in service delivery or the day-to-day running of clinical sites. As a result, some perspectives on learning as it unfolds within service-driven clinical environments may not have been fully captured. Including clinicians directly involved in patient care in future studies may enhance the transferability of findings across clinical contexts and deepen understanding of learning in the clinical environment. We recognise the unique challenges and demands experienced within the South African public health sector, and similar studies conducted in different contexts may add further layers to understanding clinical learning. Given the complexity of learning and the multiple layers of interaction between people, practices and environments, future research should continue to interrogate these layers.

One important step towards harnessing learning opportunities in the clinical environment is increasing awareness among all stakeholders of how learning is enacted. Staff development initiatives that explicitly address learning in clinical environments may support facilitators in recognising learning opportunities and in embracing the discomfort associated with shifting from a traditional ‘teacher’ role towards supporting students’ development as active, self-directed learners who navigate dual roles as learners and emerging practitioners (Dornan et al. 2009).

### Clinical implications

A key implication of these findings is the need for explicit discussion and alignment of expectations between students and facilitators. Students in this study emphasised relational safety and interpersonal engagement as central to learning, whereas facilitators more often approached learning from a system- and task-oriented perspective. Making these differing lenses explicit may reduce misalignment and support more productive learning encounters. Importantly, while students demonstrated agency in their learning, the enactment of this agency was shaped by relational safety, power dynamics and environmental constraints.

## Conclusion

Learning in the clinical environment is shaped by the people involved and how they collectively participate in learning across both implicit and explicit spaces. This study highlights that learning is not solely determined by curricular structures or clinical exposure, but by how students and facilitators understand and enact their roles within complex relational and environmental contexts.

Our findings suggest that facilitators may need to re-examine taken-for-granted assumptions about their role in clinical training, particularly in relation to recognising and supporting student agency. When opportunities for agency are constrained, students risk remaining in passive roles; when agency is acknowledged and relationally supported, learning becomes more active, adaptive and meaningful.

Clinical learning, therefore, requires a stance of curiosity and shared responsibility from all participants. Sustaining such learning environments may be supported through faculty development initiatives that foreground relational engagement, reflective facilitation and the co-creation of learning, thereby supporting the ongoing professional development of both students and educators.

## Appendix 1

Interview guide for FGDs with learners:

1. Describe your understanding of the concept ‘learning’.
2. What do you see as your role in your learning?
3. What do you see as your facilitator’s role in your learning?
4. How does teaching take place in the clinical environment?
5. How does learning take place in the clinical environment?
6. What opportunities in the clinical environment are available to you for your learning?
7. How different, if at all, is this learning from what you may have expected it to be?
8. Think of a specific teaching-learning session. Please describe the scenario.
9. Probe: What was your role in preparing for this session?
10. Probe: Do you think you could have done more, and if so, what would that be?
11. What suggestions would you make to improve learning in the learning environment?

Interview guide for FGDs/interviews with facilitators:

1. Describe your understanding of the concept ‘learning’.
2. How do you see your role in facilitating learning?
3. How does teaching take place in the clinical environments?
4. How does learning take place in the clinical environments?
5. What opportunities in the clinical environment are available for you to facilitate learning?
6. How different, if at all, is the facilitation of learning from what you may have expected it to be?
7. What do you see as the role of the learner in their learning?
8. Probe: What more would you expect them to do, and what might your role in this be?
9. What other suggestions would you make to improve the learning environment?
  - What might you do differently?
  - How might you alter the learning environment, or your support of learners, to facilitate them to develop more into independent learners?
